# Effect of a new plant-based high-energy oral nutritional supplement in adult malnourished patients: an open-label, randomized clinical trial

**DOI:** 10.3389/fnut.2025.1667954

**Published:** 2025-11-20

**Authors:** Natalia Covadonga Iglesias Hernández, Araceli Ramos Carrasco, Daniel Antonio De Luis Román, Pedro Luis de Pablos-Velasco, Alfonso Calañas Continente, Miren Josune Rodríguez Soto, Juan José López-Gómez, Cristina Comi-Díaz, Silvia García-Rey, Clara Pérez-Rambla, Pedro Pablo García-Luna

**Affiliations:** 1 Endocrinology and Nutrition Department, Basurto University Hospital, Bilbao, Spain; 2 Endocrinology and Nutrition Department, Mostoles University Hospital, Madrid, Spain; 3 University of Valladolid, Valladolid, Spain; 4 Endocrinology and Nutrition Department, University of Las Palmas de Gran Canaria, Gran Canaria, Spain; 5 Endocrinology and Nutrition Department, Reina Sofía University Hospital, Córdoba, Spain; 6 Endocrinology and Nutrition Department, Valladolid University Clinical Hospital, Health Research Institute of Valladolid (IBioVALL) and Center of Investigation Endocrinology and Nutrition, University of Valladolid, Valladolid, Spain; 7 Nutrition Unit, Endocrinology and Nutrition Department, Virgen del Rocío University Hospital, Sevilla, Spain; 8 RWE Department, Outcomes’10 SLU, Castellón de la Plana, Spain

**Keywords:** malnourished patients, oral nutritional supplement, nutritional status, plant-based diet, gastrointestinal tolerance

## Abstract

**Background and aims:**

The recent global trend toward reducing the consumption of animal-derived products has contributed to a growing demand for plant-based oral nutritional supplement (pbONS) options. PbONS, in addition to achieving good compliance, have an improvement in nutritional status (increased energy and protein intake, body weight, and BMI) on malnourished patients. However, there is no evidence of their impact compared to animal-based ONS (aONS).

**Methods:**

A multicenter, open-label, and non-inferiority trial (NCT06055543) was conducted in 5 tertiary hospitals in Spain. Adult malnourished outpatients requiring high-energy ONS for at least 12 weeks (1.5 kcal/ml;200 ml, 2 bottles/day), having signed informed consent, were recruited and randomly assigned (1:1) to receive pbONS or aONS. Randomization was performed using a pre-generated list embedded in the electronic data entry platform. The total number of participants randomized was 149, 75 patients in aONS group and 74 in pONS group. Baseline and final characteristics of the two groups were described and compared in terms of sociodemographic, clinical, compliance, and satisfaction variables.

**Results:**

A total of 129 patients who received pbONS (66) or aONS (74) were included. Weight increased significantly from 55.1 ± 12.7 kg to 57.4 ± 13.1 kg (*p*-value < 0.001) in the aONS group, and from 55.0 ± 10.7 kg to 57.1 ± 10.7 kg (*p*-value < 0.001) in the pbONS group, with no significant differences between groups (*p* = 0.659). It was further confirmed that pbONS is not inferior to aONS in terms of body weight gain. According to GLIM criteria, patients improved their nutritional status 92.1% (58/63) in the aONS group and 95.5% (63/66) in the pbONS group with no statistical difference between groups (*p*-value = 0.425). Functional strength was increased after 12 weeks: 55.6% (35/63) in aONS and 60.6% (40/66) in pbONS, with no significant difference between groups (*p*-value = 0.346). Gastrointestinal Symptom Rating Scale (GSRS) scores were lower in both groups at 12 weeks, indicating a reduction in symptom severity.

**Conclusion:**

PbONS are as effective and well-tolerated as traditional ONS in improving nutritional outcomes, with high patient satisfaction in malnourished patients. This study provides valuable evidence for integrating pbONS into routine clinical practice for the tailored management of patients.

**Clinical trial registration:**

## Introduction

Malnutrition is ‘a state resulting from lack of intake or uptake of nutrition that leads to altered body composition (decreased fat-free mass) and body cell mass, leading to diminished physical and mental function and impaired clinical outcome from disease (1). This condition can result from various factors such as starvation, aging, and disease (2, 3). Notably, 30–50% of inpatients suffer from Disease-Related Malnutrition (DRM) due to their underlying illness or associated comorbidities (4). DRM plays a critical role in disease progression, as it increases the length of hospitalization and the risk of additional comorbidities and negatively affects recovery (5, 6).

According to the guidelines and consensus statements from the European Society for Clinical Nutrition and Metabolism (ESPEN) and the American Society of Parenteral and Enteral Nutrition (ASPEN), it is essential to establish a detailed nutritional profile at the time of diagnosis of chronic diseases, particularly in oncology, in order to address malnutrition as early as possible. In this context, the parallel pathway approach has been recommended in cancer care, where nutritional screening and interventions are initiated alongside tumor diagnosis and treatment planning (7, 8). However, they also highlight the lack of standardized criteria for diagnosis of malnutrition (1, 9, 10). An internationally recognized method, the Global Leadership Initiative on Malnutrition (GLIM) criteria, has been increasingly adopted to address this limitation by providing a standardized framework that combines phenotypic and etiologic components, improving diagnostic accuracy and clinical applicability (11). Despite efforts to standardize diagnostic methods, malnutrition remains highly prevalent, with estimates suggesting that between 8–62% of patients exhibit signs of undernutrition, yet only 1.6% receive nutritional intervention (12). This gap might lead to severe consequences, particularly in oncology, where 10–20% of patient deaths are attributed to malnutrition rather than the tumor itself (13, 14).

As a therapeutic approach, current ESPEN guidelines recommend initiating nutritional counseling as the first step in managing malnutrition or nutritional risk, followed by the use of oral nutritional supplements (ONS) when appropriate. These guidelines emphasize individualized nutritional support tailored to the complexity of the patient’s condition, especially in heterogeneous populations such as polymorbid medical inpatients and older adults. Interventions should consider factors such as age, comorbidities, hydration status, and nutritional risk, ensuring that ONS use complements comprehensive clinical management (1, 9, 10). ONS have demonstrated significant clinical effectiveness in both clinical trials and real-world studies, emerging as the primary nutritional intervention for malnourished patients who are able to receive oral nutritional support (15, 16). Their benefits extend across various conditions, improving weight, nutritional status, and muscular strength while reducing hospital stays, the risk of comorbidities, and healthcare costs (16, 17). However, their effectiveness largely depends on patient adherence (18). Adherence to ONS has been heterogeneously reported in the literature (19), with suboptimal adherence rates often due to issues such as flavor fatigue or gastrointestinal intolerance (20). To address these challenges, a wide range of formulations with different flavors and nutritional compositions have been developed to provide personalized therapy, enhance patient satisfaction, and improve adherence, ultimately increasing the efficacy of the intervention (21). However, there is still a significant lack of plant-based supplements, with most currently available ONS formulations containing cow’s milk or other animal-derived ingredients (22, 23). Developing and validating these alternatives is essential to meet patient preferences and address specific health-related concerns. It is estimated that 75% of the population exhibits some degree of lactose intolerance (24), and a recent meta-analysis revealed a prevalence of 0.6% for cow’s milk protein allergy (25), so affected individuals are left with limited or unsuitable alternatives. Moreover, approximately 5% of the European population identifies as vegetarian (26), and 23% aim to reduce their consumption of animal-based products (27). As a result, the demand for plant-based nutritional products is expected to grow significantly in the coming years (28).

Within this framework, the present study, designed as a non-inferiority randomized clinical trial, aimed to evaluate the efficacy of a plant-based ONS compared to standard ONS in patients who were malnourished or at risk of malnutrition. The control product was a dairy-based supplement with equivalent energy and protein content. Additionally, this study incorporated patient perspectives to assess satisfaction with these ONS, aiming to determine whether they represent an effective and valuable therapeutic alternative for routine clinical practice.

## Materials and methods

### Study design

This multicenter, randomized, open-label trial was conducted at five tertiary hospitals in Spain. The study was registered at clinicaltrials.gov (NCT06055543), conducted in accordance with the principles of the Declaration of Helsinki (29), approved by Euskadi Ethics Committee (CEIm-E; intern code PI2023069) on May 10, 2023, and reported according to the CONSORT statement. Before inclusion, all patients provided written informed consent. The study was conducted as an open-label trial, as the two ONS differed in taste and appearance, making blinding unfeasible. Although blinding was not feasible, and the characteristics of the supplements may have influenced certain parameters such as patient satisfaction and treatment compliance, the primary endpoints of this study relied on objective assessments, including anthropometric measurements and physician-evaluated parameters such as nutritional screening and evaluation. Patient perspectives served to complement the overall analysis. All patients were fully informed and provided consent.

### Recruitment and study participants

The recruitment period was from September 2023 to March 2024. Outpatients were eligible for inclusion in the trial if they were 18 years of age or older, presented malnutrition according to the Global Leadership Initiative on Malnutrition (GLIM) criteria (11); had a high-energy ONS requirement (1.5 kcal/ml) as a part of their nutritional support plan, with an expected intake of at least two bottles of ONS per day for at least 12 weeks; and presented any of the following clinical situations: (i) elderly patients requiring oral nutritional support; (ii) digestive pathology including but not limited to inflammatory bowel disease, short bowel syndrome, pancreatitis, without active malabsorption and maldigestion; (iii) chronic disease, such as chronic obstructive pulmonary disease (COPD), mild to moderate renal disease, positive for Human immunodeficiency virus (HIV); (iv) oncologic patients with an Eastern Cooperative Oncology Group (ECOG) performance status of 0–1. Patients were excluded if they needed ONS due to surgery or acute illness, with known intolerance or allergy to ingredients of the study products, with hyperthyroidism or uncontrolled hypothyroidism, uncontrolled diabetes (HbA1c > 8%), requiring enteral tube feeding, or ostomy nutrition, with moderate or severe renal insufficiency, or with abnormal hemoglobin or transferrin laboratory values.

### Randomization and allocation

Following enrollment, patients were randomly assigned in a 1:1 ratio to either the intervention or control group. Randomization was performed using a pre-generated simple randomization list embedded in the electronic data entry platform. When a new participant was entered into the system, group assignment was automatically provided according to the list. Allocation was not concealed from researchers or participants. Patients in the intervention group received a plant-based ONS (pbONS), specifically Fortimel PlantBased ® 1.5 kcal/ml, Nutricia Ltd., Spain, while those in the control group received the standard animal-based ONS formulation (aONS), specifically Fortimel ® 1.5 kcal/ml, Nutricia Ltd., Spain (product data sheet in Supplementary Table 1). Fortimel Original contains casein as its only protein source, while Fortimel PlantBased contains proteins derived from soy and pea. Both ONS are lactose-free (PbONS <0,025 g lactose/100 ml vs. aONS = 0 g lactose/100 ml), high-energy formulations (1.5 kcal/ml, 12 g protein per 200 ml serving), and participants were prescribed two 200 ml bottles daily for at least 12 weeks (product characteristics are detailed in Supplementary Table 1). Variables were collected in an electronic case report form comprising two modules; one completed by the research team and the other by the patient/caregiver (Table 1).

**Table 1:** Variables and time points assessed.

Outcome	Baseline	Final visit (Day 85)
Sociodemographic and clinical variables, diagnosis and comorbidities	HCP	-
MUST	HCPs	HCPs
GLIM	HCPs	HCPs
Handgrip	HCPs	HCPs
Calf circumference	HCPs	HCPs
GSRS	Patients	Patients
Laboratory parameters	HCPs	HCPs
Compliance^*^	-	Patients/Caregivers
Satisfaction	-	Patients

GLIM, global leadership initiative on malnutrition; GSRS, gastrointestinal symptoms rating scale; HCP, healthcare professionals, MUST: malnutrition universal screening tool; *Variables recorded every day during the intervention period.

### Data collection and study variables

Prior to study initiation, patient demographics variables (age, gender) and clinical data (main diagnosis and comorbidities) were extracted from medical records.

#### Assessment of body weight and nutritional status

Weight gain percentage was determined by calculating the relative change in body weight from baseline to week 12. To assess improvements in nutritional risk, physicians recorded the MUST score and GLIM criteria for patients. The MUST questionnaire was employed to evaluate the risk of malnutrition (30). The total score reflects the overall risk of malnutrition: score = 0, low risk; score = 1, medium risk; and score ≥2, high risk. Changes in the proportion of patients at each level of malnutrition risk were assessed by comparing baseline and final visit scores.

The GLIM criteria were employed to evaluate changes in nutritional status (11). This tool scores on a scale of 0 to 2 to define malnutrition, where higher scores account for a higher malnutrition status. Changes in nutritional status were evaluated by comparing baseline and final visit evaluation scores, a shift from grade 2 (severe) to grade 1 (moderate) representing an improvement, or from grade 1 to 2, failure to meet GLIM criteria.

#### Assessment of the functional status

To determine changes in functional status, the difference in handgrip strength (kg/cm^2^) from baseline to the final visit was calculated using a dynamometer. Patients were classified into three groups: strength gain (difference in strength >0), strength maintenance (difference in strength = 0), and strength loss (difference in strength <0).

To assess changes in calf circumference (cm) as a measure of muscle status, measurements taken at baseline and the final visit were compared. Patients were classified into three groups according to the difference between the adjusted baseline and final measurements: improvement (difference > 0), maintenance (difference = 0), and no improvement (difference < 0). To account for differences related to gender and BMI, calf circumference values were adjusted (as a variable “adjusted calf circumference”) by applying correction factors based on the methodology specified by Gonzalez MC et al., in (31).

#### Assessment of the gastrointestinal symptoms

To evaluate gastrointestinal symptoms, the Gastrointestinal Symptom Rating Scale (GSRS) (32) scores were calculated. This test consists of 15 items grouped into five symptom clusters: reflux, abdominal pain, indigestion, diarrhea, and constipation. Each item is rated on a 7-point Likert scale by the patient, and a domain-specific GSRS score is calculated as the average of subitems. Changes in the scores for each domain were evaluated by comparing the baseline and final visit values. The scores range go from 0 indicating “no discomfort” to 7 “very severe.”

#### Assessment of patient compliance with ONS

To assess compliance with oral nutritional supplement (ONS) intake, patients or their caregivers completed a daily electronic consumption diary (recording the amount wasted per serving). The diary was accessed through a digital platform that was also available to the investigator, who monitored adherence according to their clinical judgment, in line with the study’s design as routine clinical practice. The amount wasted was measured using standardized containers provided by the study sponsor. (The number of bottles consumed and not consumed was also recorded.) Compliance was calculated as the percentage of bottles consumed out of the total number provided (168 bottles over 84 days, at two bottles per day). At the final visit, the investigator reviewed the recorded data with the patient to identify and resolve any discrepancies.

Finally, patient satisfaction with the ONS was assessed on the final visit using an *ad hoc* questionnaire. The questionnaire comprised three modules. In the first module, a visual analog scale (VAS) ranging from 0 to 10 (0 = “nothing” and 10 = “a lot”) was used to rate the patient’s overall satisfaction with the ONS, satisfaction with the flavor, and the ease of taking the supplement. In the second module, patients answered “yes” or “no” to determine whether they felt satiated after taking ONS. In the final module, patients indicated the extent to which feeling satiated affected their regular daily intake, selecting one of the following options: “a lot,” “quite a lot,” “somewhat,” or “not at all.”

Additionally, changes in laboratory variables from baseline to the final visit were also calculated. These variables included albumin, prealbumin, Zinc, Hemoglobin, c-reactive protein (CRP), Ferritin, Transferrin, Hemoglobin A1c (HbA1c), Creatinine, thyroid-stimulating hormone (TSH), glomerular filtration rate (GFR), and Lymphocytes.

### Sample size calculation

The sample size for this study was estimated based on a 5.2 ± 5.9% body weight gain observed with high-energy ONS detailed in (16), a non-inferiority margin of 2.5% (*δ*), a 90% one-sided confidence level (zα = 1.645); and a statistical power of 80% (zβ = 0.842). The result of this calculation was 140 patients (70 per group). Based on the same assumptions, statistical power was calculated at the conclusion of the study, considering a sample size of 63 patients per group. Final sample size yielded 77.1% statistical power due to 18 dropouts.

### Statistical analysis

Data analysis was planned and performed for two data sets: by intention-to-treat and by protocol. However, the analysis was shown according to protocol, as the sample size and results were similar in both data sets. Intention-to-treat results are shown in Supplementary Figure 1. Non-inferiority in weight gain between the two study groups was assessed by examining the lower limit of the 90% confidence interval for the difference in weight gain between the two study arms. The non-inferiority margin was set at *δ* = 2.5%, and inferiority was determined if the lower confidence interval limit exceeded this threshold. This margin was based on preserving 50% of the mean body weight gain reported in previous studies (16), to ensure retention of a clinically meaningful proportion of the treatment effect.

Relative and absolute frequencies were calculated to describe qualitative variables. Central tendency and dispersion measures (mean, standard deviation, quartiles, minimum, and maximum) were computed for quantitative variables. Normality was assessed using the Shapiro–Wilk test. A descriptive analysis was conducted for both baseline and final visit variables. The difference between these time points was calculated for each variable and categorized as an increase, the same, or a decrease. Differences between the aONS and pbONS groups were assessed using the non-parametric Mann–Whitney U test for quantitative variables and the Chi-square test for qualitative variables, considering a significant level of 5%.

Additionally, baseline variables were compared between groups using the Mann–Whitney U test for quantitative variables and the Chi-square test for qualitative variables, with a significance level set at 5%. Moreover, differences between baseline and final visit values for the main variables (weight, GLIM, and MUST score) were calculated for each ONS using Wilcoxon Signed-Rank and McNemar’s Chi-squared test, considering a significant level of 5%. Statistical analyses were performed using the STATA v.14 software (College Station, Texas, USA).

## Results

### Sociodemographic and clinical characteristics of the study population

**Figure 1:**
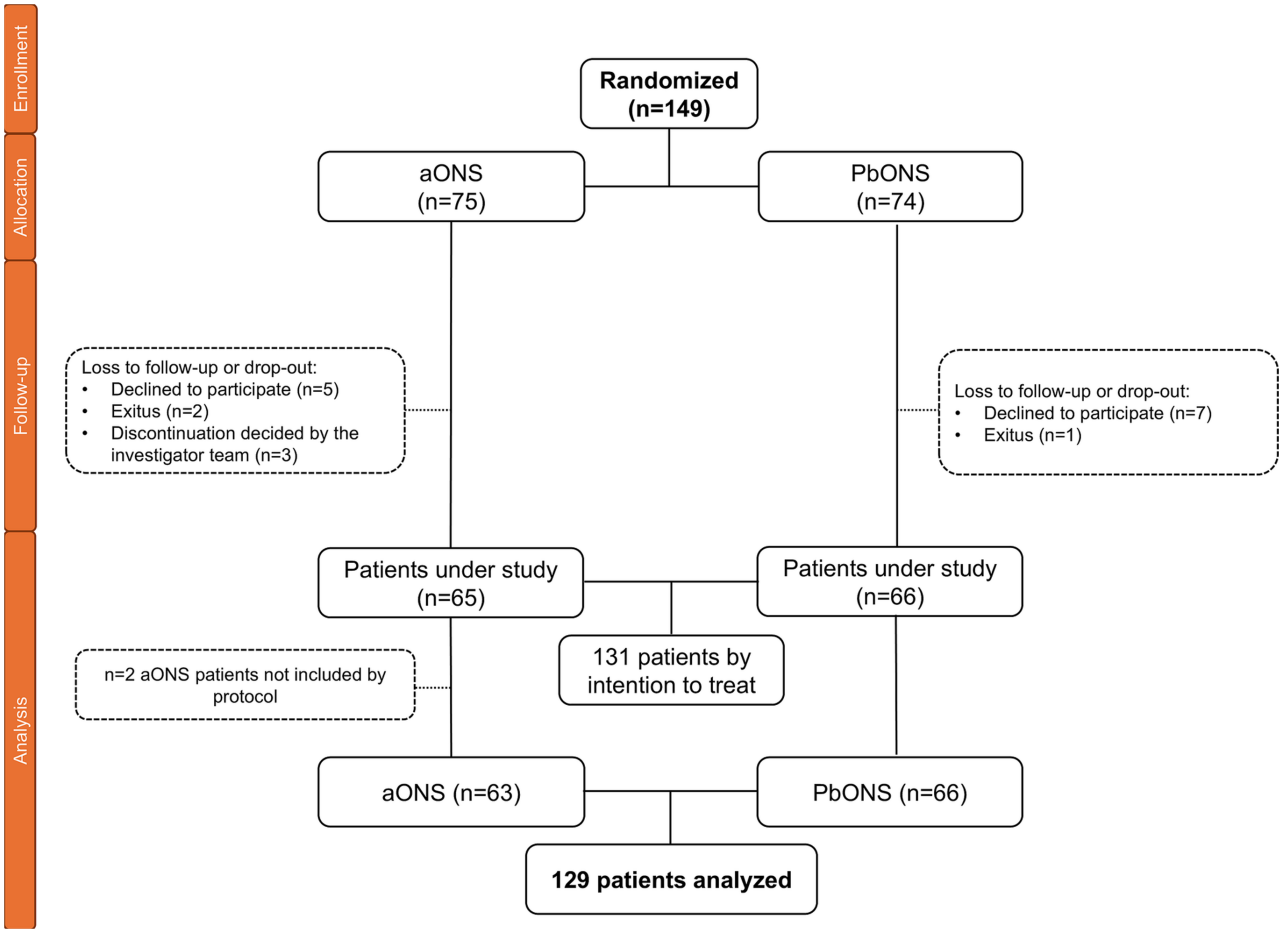
Consort flow diagram.

Of the total number of 129 patients included in the study, 63 patients received aONS and 66 patients received pbONS. Final sample size yielded 77.1% statistical power due to 18 dropouts (Figure 1). The mean ± SD age was 60.7 ± 16.4 years, 54.3% (70/129) of patients were male, and among clinical conditions associated with malnutrition, chronic diseases were the most common (37.2%, 48/129), with cancer being the most prevalent diagnosis (31.0%, 40/129) (Table 1).

The mean ± SD body weight at baseline was 55.1 ± 11.7 kg. According to the MUST score, 74.4% (96/129) of patients were at high risk of malnutrition, while 24.0% (31/129) were at medium risk.

At baseline, no statistically significant differences were found in the main variables between the two groups of patients (Table 2).

**Table 2:** Sociodemographic and clinical characteristics of patients in both groups.

Nutritional characteristics at baseline and at week 12			
Variable number of patients, *n*	Total 129	aONS 63	PbONS 66
Age in years, mean ± SD	60.7 ± 16.4	58.6 ± 15.9	62.7 ± 16.7
Gender			
Male, *n* (%)	70 (54.3)	31 (49.2)	39 (59.1)
Female, *n* (%)	59 (45.7)	32 (50.8)	27 (40.9)
Clinical situation related to malnutrition, n (%)			
Chronic disease^*^	48 (37.2)	25 (39.7)	23 (34.9)
Elderly patient requiring ONS	9 (7.0)	2 (3.2)	7 (10.6)
Digestive pathology without active malabsorption/maldigestion	35 (27.1)	18 (28.6)	17 (25.8)
Oncological patients with ECOG 0–1	37 (28.7)	18 (28.6)	19 (28.8)
Main diagnosis, *n* (%)			
Cancer	40 (31.0)	20 (31.8)	20 (30.3)
COPD	22 (17.1)	10 (15.9)	12 (18.2)
Crohn’s Disease	8 (6.2)	4 (6.4)	4 (6.1)
Irritable bowel syndrome	8 (6.2)	4 (6.4)	4 (6.1)
Ulcerative colitis	4 (3.1)	1 (1.6)	3 (4.6)
Pancreatitis	3 (2.3)	2 (3.2)	1 (1.5)
HIV	1 (0.8)	0 (0.0)	0 (0.0)
Other	43 (33.3)	21 (33.3)	22 (33.3)
Comorbidities^**^, *n* (%)			
Gastrointestinal disease	37 (28.7)	12 (19.1)	25 (37.9)
Cardiovascular disease	36 (27.9)	15 (23.8)	21 (31.8)
Endocrine, Nutritional, or Metabolic Disease	24 (18.6)	6 (9.5)	18 (27.3)
Musculoskeletal disease	15 (11.6)	8 (12.7)	7 (10.6)
Visual or hearing disease	14 (10.9)	8 (12.7)	6 (9.1)
Infectious or Parasitic disease	10 (7.8)	3 (4.8)	7 (10.6)
Other	10 (7.8)	5 (7.9)	5 (7.6)
Height in cm, mean ± SD	164.7 ± 9.5	165.0 ± 10.3	164.4 ± 8.8
Body weight in kg, mean ± SD	55.1 ± 11.7	55.1 ± 12.7	55.0 ± 10.7

	Total		aONS		pbONS	
	BL	FV	BL	FV	BL	FV
Global risk of malnutrition (based on MUST Score^***^), *n* (%)						
High risk	96 (74.4)	41 (31.78)	45 (71.4)	20 (31.8)	51 (77.3)	21 (31.8)
Medium risk	31 (24.0)	23 (17.83)	17 (27.0)	13 (20.6)	14 (21.2)	10 (15.2)
Low risk	2 (1.6)	65 (50.39)	1 (1.6)	30 (47.6)	1 (1.5)	35 (53.0)
GLIM Phenotypic criteria, mean ± SD						
BMI (kg/m^2^)	20.3 ± 3.7	21.07 ± 3.75	20.1 ± 3.9	21.02 ± 3.93	20.4 ± 3.6	21.12 ± 3.60
Calf circumference in cm	30.0 ± 3.9	31.48 ± 3.88	30.5 ± 4.1	32.18 ± 4.00	29.6 ± 3.7	30.82 ± 3.67
Adjusted calf circumference	30.9 ± 3.3	31.93 ± 3.34	31.3 ± 3.3	32.85 ± 3.19	30.4 ± 3.3	31.06 ± 3.27
ASMI^****^	5.4 ± 1.6	5.84 ± 1.56	5.4 ± 1.6	5.85 ± 1.58	5.5 ± 1.7	5.82 ± 1.56
Etiological criteria, *n* (%)						
Reduced food intake or assimilation	79 (61.2)	12 (8.94)	40 (63.5)	5 (7.94)	39 (59.1)	7 (10.61)
Inflammation	103 (79.8)	66 (51.16)	48 (76.2)	34 (53.97)	55 (83.3)	32 (48.48)
GLIM						
Moderate malnutrition	46 (35.7)	13 (10.08)	23 (36.5)	8 (12.70)	23 (34.9)	5 (7.58)
Severe malnutrition	83 (64.3)	2 (1.55)	40 (63.5)	1 (1.59)	43 (65.2)	1 (1.52)
Handgrip strength in kg/cm^2^, mean ± SD						
	24.3 ± 9.7	25.51 ± 10.43	25.7 ± 10.0	26.72 ± 10.28	22.9 ± 9.2	24.36 ± 10.52
Calf circumference in cm, mean ± SD						
	30.0 ± 3.9	31.48 ± 3.88	30.5 ± 4.1	32.18 ± 4.00	29.6 ± 3.7	30.82 ± 3.67
Adjusted calf circumference in cm, mean ± SD						
	30.9 ± 3.3	31.93 ± 3.34	31.3 ± 3.3	32.85 ± 3.19	30.4 ± 3.3	31.06 ± 3.27

aONS, animal-based ONS; ASMI, appendicular skeletal muscle index; BL, baseline; BMI, body mass index; FV, final visit; GLIM, global leadership initiative on malnutrition; MUST, malnutrition universal screening tool; ONS, oral nutritional supplement; pbONS, plant-based high energy ONS; SD, standard deviation.

* Chronic disease such as chronic obstructive pulmonary disease (COPD), mild to moderate renal disease, and individuals positive for Human Immunodeficiency Virus (HIV).

** Subjects could have more than one comorbidity, and the percentages do not add up to 100%.

*** MUST scores were calculated by combining BMI, weight loss, and acute disease effect. Categories are defined as: 0 = Low risk (routine clinical care), 1 = Medium risk (observe), and ≥2 = High risk (treat).

**** ASMI: Calculated by using: ASMI_v1 = (−10.427 + calf_circ_v1 × 0.768 − age × 0.029 + (gender − 1) × 7.523)/height^2^ (m).

### Changes in body weight

**Figure 2:**
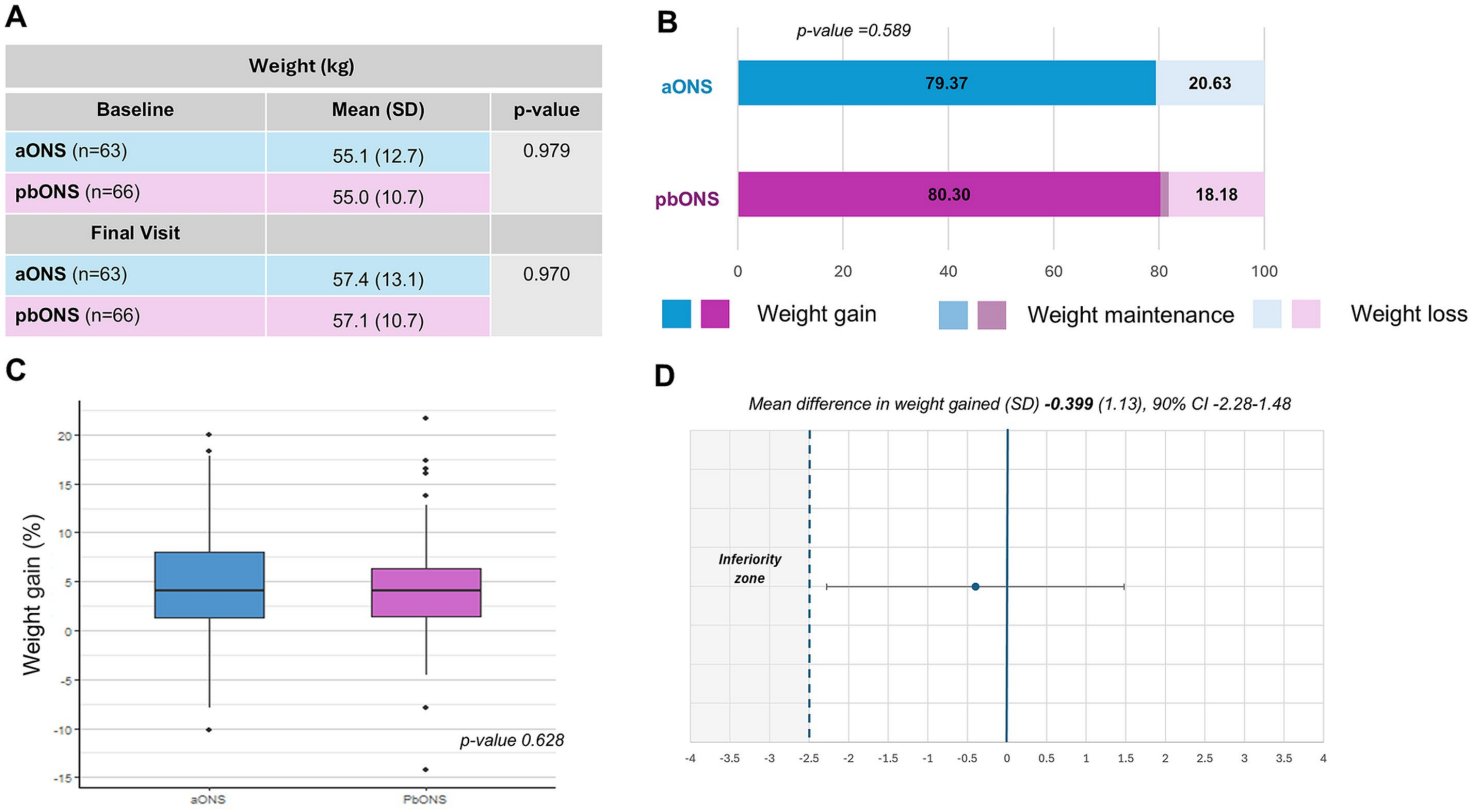
Effects of ONS on body weight changes. **(A)** Body weight data by group, differences at Baseline and at Final Visit; **(B)** Body weight improvement; **(C)** Box-plot of body weight gain (%); **(D)** Non-inferiority in weight gain between the two study groups.

The mean ± SD weight gain from baseline to final visit was 2.3 ± 3.8 kg in the aONS group and 2.1 ± 3.4 kg in the pbONS group, with no significant differences between groups (*p* = 0.659). The differences between groups were also not significant at baseline or at the final visit (Figure 2A). Weight gain was observed in 79.4% (50/63) of aONS patients and 80.3% (53/66) of pbONS patients (Figure 2B). A mean ± SD percentage increase of 4.5 ± 6.9% was observed in aONS and of 4.1 ± 5.9% in pbONS (Figure 2C). As shown in Figure 2D, the lower limit of the 90% one-sided confidence interval for the difference between groups remained within the predefined non-inferiority margin, confirming that pbONS is not inferior to aONS in terms of body weight gain. The mean difference between groups was −0.399 (SD = 1.13), with a 90% two-sided confidence interval of [−2.28 to 1.48]. The results by intention-to-treat population are shown in Supplementary Figure 1.

For each product, a significant difference (*p* < 0.001) was observed in the mean ± SD weight gain from baseline to the final visit. In the aONS group, the mean weight increased from 55.1 ± 12.7 kg at baseline to 57.4 ± 13.1 kg at the final visit. In the pbONS group, the mean weight increased from 55.0 ± 10.7 kg to 57.1 ± 10.7 kg. The *p*-values for the changes in key variables from baseline to the final visit for each product are detailed in Supplementary Table 2.

### Assessment of improvements in nutritional status

**Figure 3:**
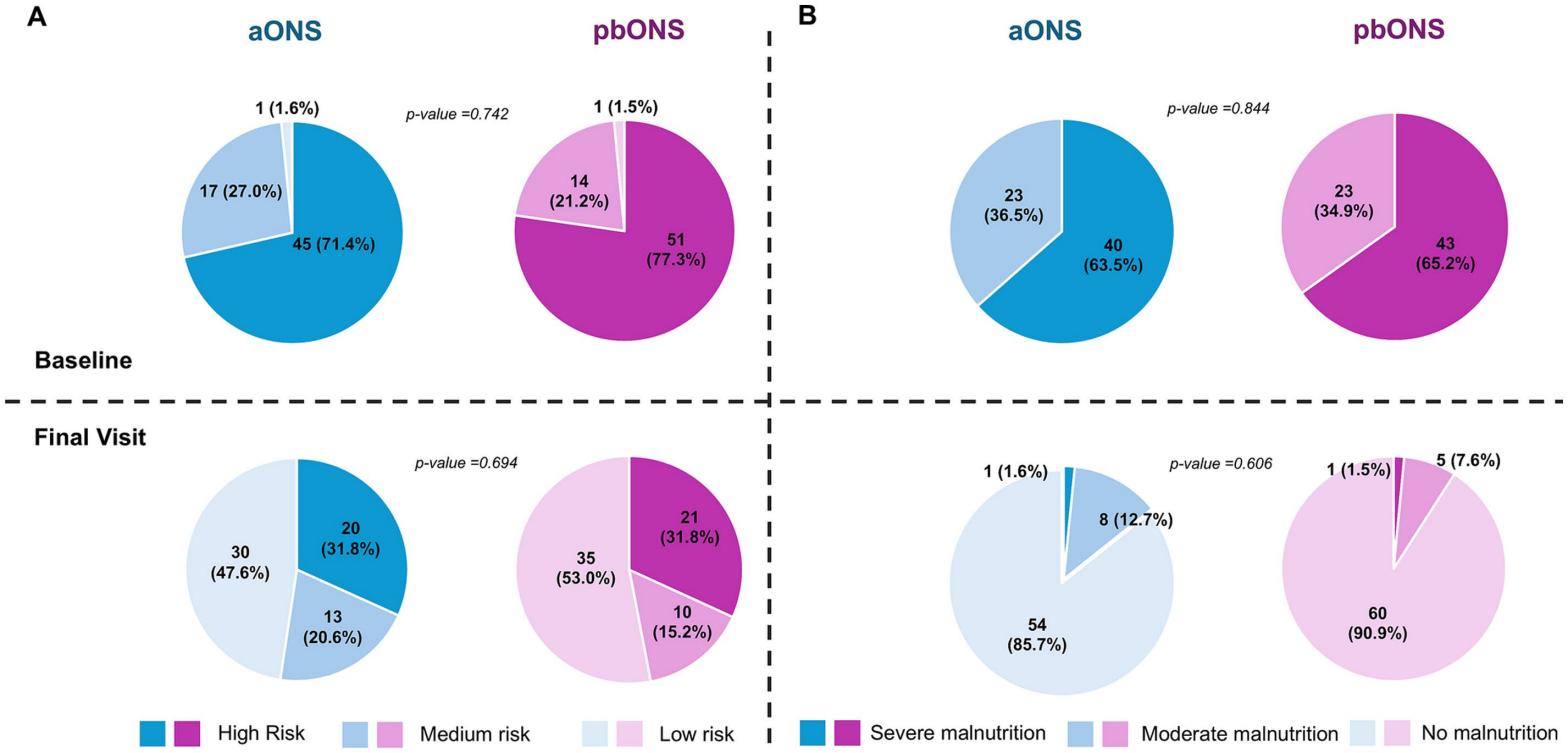
Changes in nutritional status according to MUST and GLIM scores. **(A)** Global risk of malnutrition (MUST); **(B)** Global Leadership Initiative on Malnutrition (GLIM).

After 12 weeks of ONS supplementation, according to MUST score (Figure 3A), the proportion of patients classified as low risk significantly increased in both groups, rising from 1.6% (1/63) at baseline to 47.6% (30/63) at the final visit in the aONS group (*p*-value <0.001) and from 1.5% (1/66) to 53.0% (35/66), respectively, in the pbONS group (p-value <0.001). Overall, 79.4% (50/63) of aONS group and 86.4% (57/66) of pbONS group showed an improvement in malnutrition risk, with no statistically significant differences between groups (*p*-value = 0.447). The proportion of patients with severe malnutrition significantly decreased in both groups (*p*-value <0.001 in each ONS), from 63.5% (40/63) in the aONS group and 65.2% (43/66) in the pbONS group at baseline to 1.6% (1/63) and 1.5% (1/66), respectively, at the end of the study according to GLIM (Figure 3B). By week 12, most patients showed no malnutrition: 85.7% (54/63) in the aONS group and 90.9% (60/66) in the pbONS group. Overall, 92.1% (58/63) of aONS patients and 95.5% (63/66) of the pbONS patients improved their nutritional status, with no statistical difference between groups (*p*-value = 0.425).

### Assessment of muscle mass and functional status

**Figure 4:**
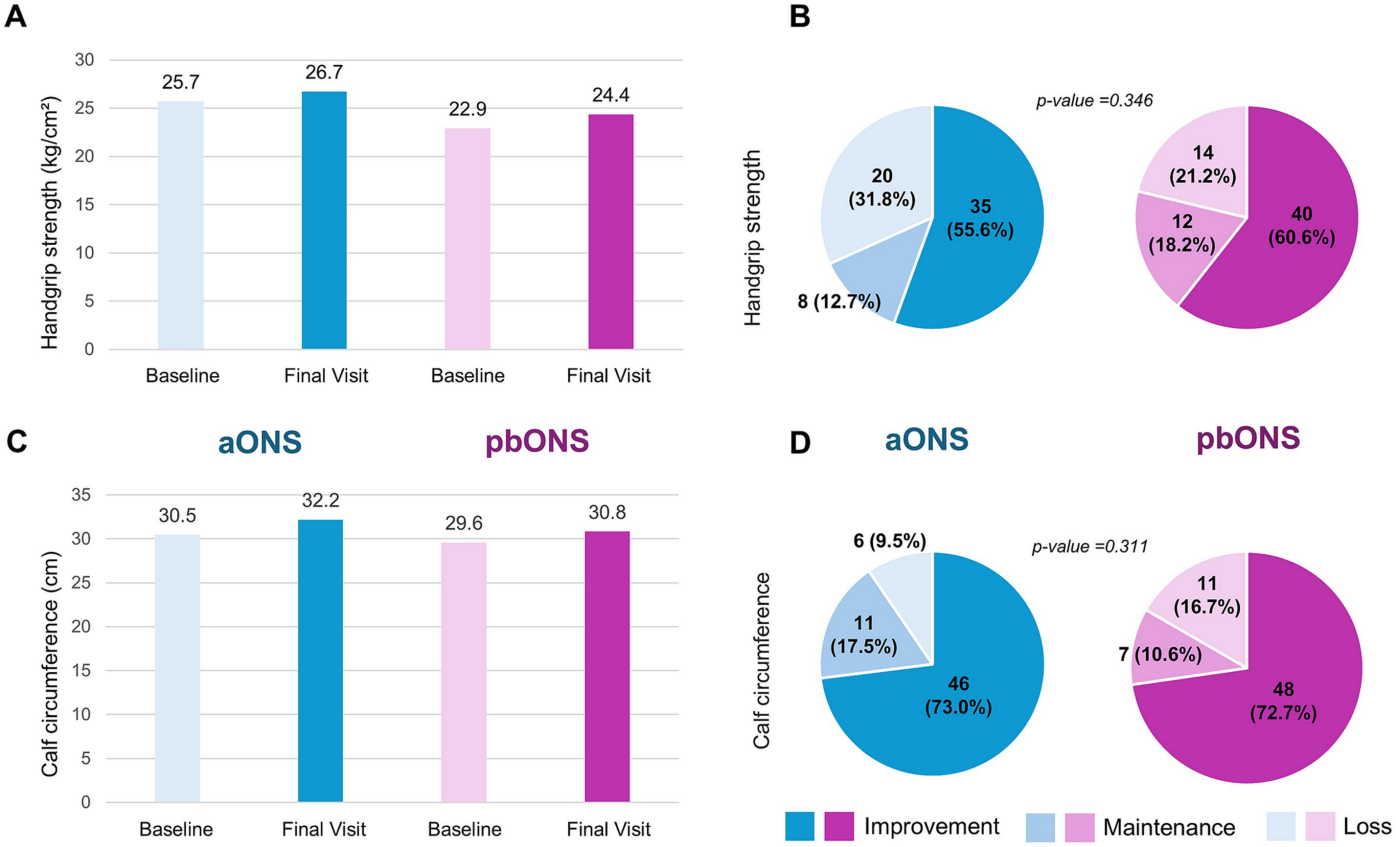
Analysis of the functional improvement and the muscle mass. **(A)** Mean value of handgrip strength; **(B)** Handgrip strength improvement; **(C)** Mean value of calf circumference; **(D)** Calf circumference improvement.

Compared to baseline, both groups showed increased handgrip strength: 1.0 ± 4.0 kg/cm^2^ for patients receiving aONS (*p*-value = 0.047) and 1.5 ± 4.4 kg/cm^2^ for patients receiving pbONS (p-value = 0.003); with no significant difference between groups (*p*-value = 0.815) Figure 4A. Our results indicated that 55.6% (35/63) of patients receiving aONS and 60.6% (40/66) of those receiving pbONS experienced an increase in functional strength after 12 weeks of ONS supplementation, with no significant difference between groups (p-value = 0.346) (Figure 4B). Both groups showed increased calf circumference (1.7 ± 2.3 cm for patients receiving aONS and 1.2 ± 2.5 cm for patients receiving pbONS, p-value <0.001), with no significant difference between groups (p-value = 0.24) (Figure 4C). Notably, 73.0% (46/63) of patients receiving aONS and 72.7% (48/66) of patients receiving pbONS demonstrated an increase in calf circumference, with no significant differences between groups (*p*-value = 0.311) (Figure 4D).

### Assessment of gastrointestinal tolerance

**Figure 5:**
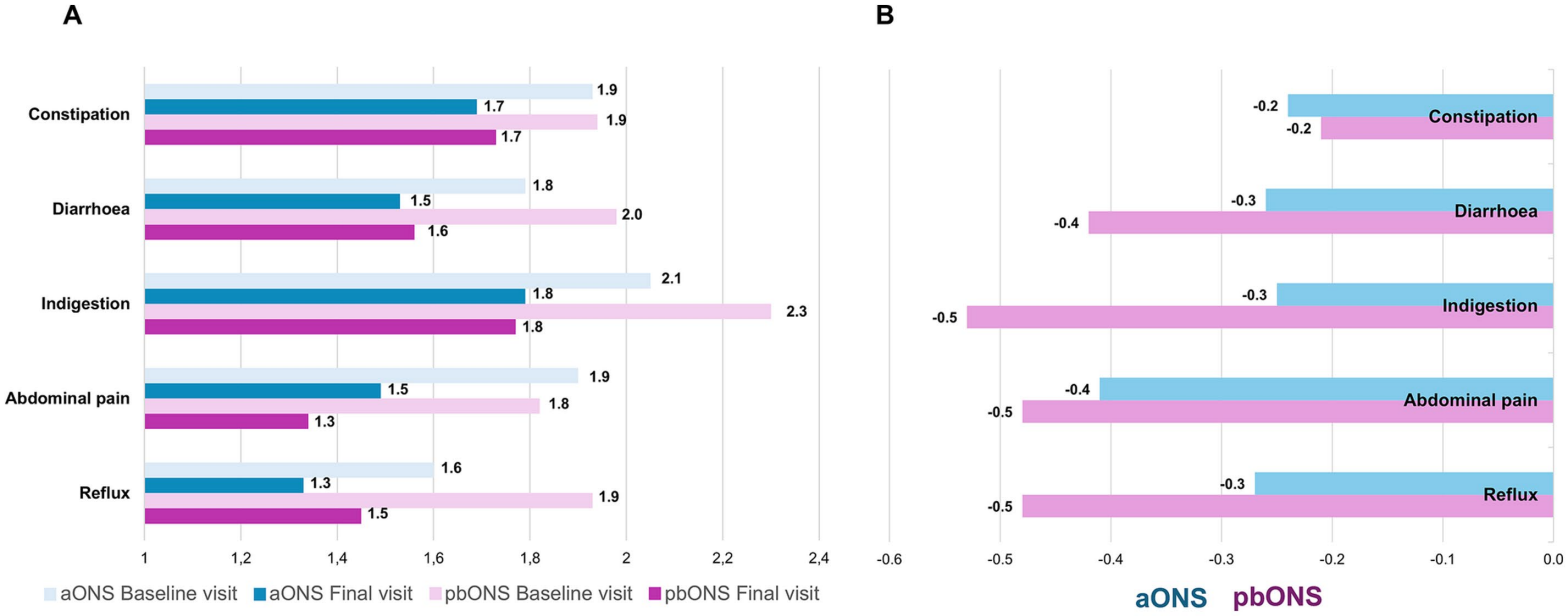
Gastrointestinal symptoms. **(A)** Mean score of each domain of the GSRS; **(B)** Change from baseline to final visit in GSRS score.

The total GSRS score was 28.24 ± 10.13 in aONS and 30.29 ± 14.23 in pbONS at baseline, while it was 23.97 ± 9.75 in aONS and 23.85 ± 8.78 in pbONS at the final visit. All the GSRS domains showed mean scores around 2 points in both groups at baseline and the final visit (Figure 5A). Scores at 12 weeks were consistently lower in both groups, indicating a reduction in symptom severity (Figure 5B) and a trend towards better tolerance (reduced reflux, diarrhea, and indigestion) with pbONS, however, without a significant difference between pbONS and aONS (*p*-value = 0.58, *p* = 0.37, and *p* = 0.40, respectively).

### Patients’ compliance with ONS

Considering that the total number of bottles provided to each patient was 168, and the maximum per day was 400 ml, patients receiving aONS consumed a mean ± SD of 363.5 ± 56.2 ml per day, while pbONS patients consumed 330.8 ± 88.6, with significant differences between groups (*p*-value = 0.026).

The observed mean compliance with ONS intake was above 80% in both groups (90.9 ± 14.1% in aONS vs. 82.7 ± 22.2% in pbONS), showing statistically significant differences between groups (p-value = 0.029).

### Patients’ satisfaction with ONS

**Figure 6:**
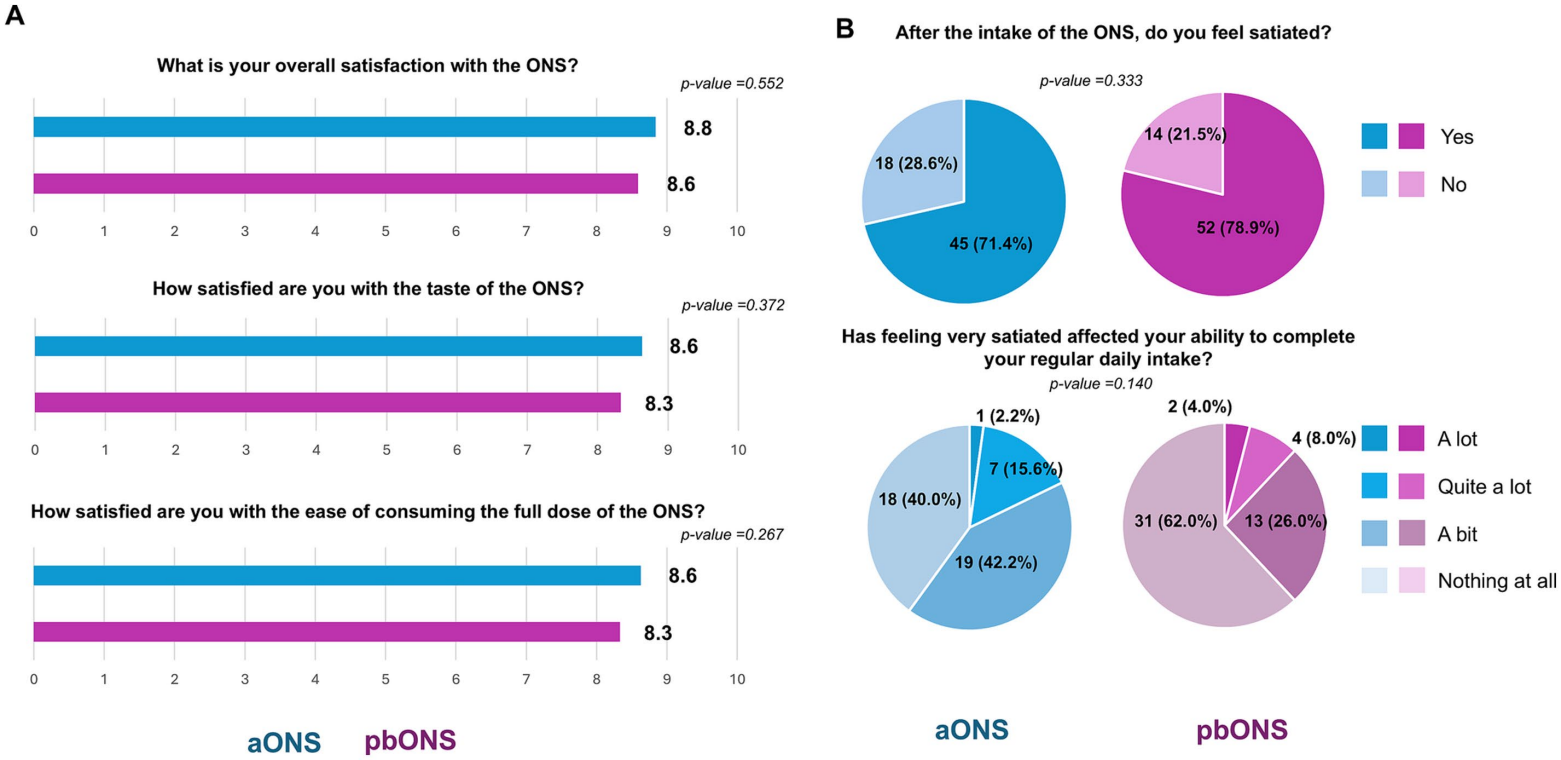
Satisfaction (ad hoc questionnaire) with the given ONS. **(A)** Satisfaction-related items; **(B)** Satiety-related items.

Overall satisfaction with the product was high in both groups (p-value = 0.552), with mean ± SD scores of 8.8 ± 1.7 points for aONS patients and 8.6 ± 2.0 points for pbONS patients (Figure 6A). High satisfaction scores were also reported for ONS taste (8.6 ± 2.0 for aONS and 8.3 ± 2.1 for pbONS, p-value = 0.372), and ease of consumption (8.6 ± 1.9 for aONS and 8.3 ± 2.1 for pbONS, *p*-value = 0.2665), with no statistically significant differences between groups. Most patients felt satiated after taking the ONS (71.4% (45/63) for aONS and 78.8% (52/66) for pbONS), and more than 80% of patients reported that feeling very satiated did not, or only slightly, affect their ability to eat their regular meals, with no significant differences between groups (Figure 6B).

### Assessment of laboratory variables

Changes in laboratory parameters following ONS supplementation were also assessed. A descriptive analysis of these variables is detailed in Supplementary Table 3 and reflects that those patients treated with pbONS present comparable biochemical parameters to those observed in aONS-treated patients.

## Discussion

### Current needs

To date, this is the first comparative study assessing the impact of both animal-based and plant-based ONS on patients´ nutritional status while examining functional capacity and muscle mass. In recent years, several authors have emphasized the need for plant-based nutritional strategies not only to meet patient demands but also to accommodate individuals with dietary intolerances and food allergies that limit the use of traditional nutritional supplements (10, 22–24, 33). There is also growing demand for environmentally sustainable alternatives to animal-based products. In clinical nutrition, sustainability refers to dietary solutions that not only meet clinical needs but also reduce environmental impact, such as greenhouse gas emissions and resource use, and remain feasible, acceptable, and accessible in diverse patient populations. However, the development and clinical testing of plant-based ONS remain limited.

### General description

Our findings substantiate that pbONS achieve comparable (*p* = 0.659) results to aONS, significantly increasing body weight, and suggesting an improved function (*p*-value = 0.346), and muscle mass (p-value = 0.24) while also being well-tolerated and highly rated by patients. To comprehensively assess the effects of pbONS, a study with broad inclusion criteria was designed to capture a wide range of clinical situations leading to malnutrition, as well as patients with different chronic diseases or older individuals who are candidates for ONS in routine clinical practice. However, the results should not be generalized to the entire population and may be more applicable to specific clinical contexts or patient profiles.

### Baseline characteristics

Firstly, both study groups exhibited similar baseline characteristics. The mean age of our cohort (60.7 years ± 16.4 years) is consistent with real-world studies evaluating ONS (23, 34), also accounting for a sex-balanced distribution. Additionally, a high percentage of patients in our study had chronic diseases (37.2%), which are strongly associated with the development of disease-related malnutrition (1). This factor underlines the consistent beneficial effects of ONS on body weight gain in chronically ill patients described in the literature (35). Nearly one-third of the study population comprised oncological patients, a proportion slightly higher than the 21% reported in a recent real-world study evaluating the efficacy of pbONS (23). Both ESPEN and the European Society for Medical Oncology (ESMO) guidelines strongly recommend ONS in patients with cancer since malnutrition in these patients is common due to the tumor or associated comorbidities (36, 37). This is particularly evident in cases where tumor location impairs food intake (38, 39). Additionally, cancer treatments frequently alter the patient’s sense of taste and smell, further compromising proper nutrition (40). A systematic literature review and meta-analysis has corroborated the beneficial effects of these supplements in promoting body weight gain in patients with cancer (mean difference, +1.3 kg). Patients in our cohort reported a broad spectrum of comorbidities and a high rate of inflammation (79.8%). This latter parameter is particularly relevant, as inflammation impairs the efficacy of nutritional interventions (41). All these characteristics highlight our cohort of patients as a representative example of real-world patients.

### Body weight, nutritional and functional status changes

In this work, ONS supplementation led to a mean body weight increase of approximately 2 kg in both groups, within the range reported in previous findings, such as the 1.7 kg gain described in the meta-analysis of ONS usage (42), the 1.3 kg gain reported among old malnourished people (43), and the 2.6 kg gain evidenced with the use of similar high-calorie ONS (23). Besides, the percentages of weight gain observed here (4.5 and 4.1%) and the proportion of patients who gained body weight (79.4 and 80.3%) closely align with the values reported in the aforementioned study including elderly patients (5% of body weight gain and 90.2% of patients gaining weight) (43). Notably, the 2.1 kg increase observed with pbONS exceeds the results of Delsoglio et al., which reported a significant but lower body weight gain of 0.6 kg, although their time of intervention was shorter (23). The non-inferiority analysis conducted in our study confirms that, in terms of body weight gain, pbONS is not inferior to aONS, supporting its use as an effective strategy to improve nutritional status. However, results should be cautiously extrapolated as the confidence interval nearly reached the limit. Body weight changes were further validated by evaluating the nutritional status and malnutrition risk using the GLIM criteria. The results regarding nutritional status observed in this study are particularly striking, with improvements in approximately 90% of patients. The substantial significant reduction in malnutrition prevalence in both groups, with an improved nutritional status in 92.1% of aONS patients (*p*-value = 0.009) and 95.5% of pbONS patients (*p*-value = 0.008), exceeds the 28% reduction in malnutrition status reported in a 3-month observational study evaluating ONS in patients with diabetes mellitus (44).

A strength of our study is the inclusion of a functional assessment based on handgrip strength, following ESPEN recommendations to detect sarcopenia (1) and the use of dynamometry as a sensitive method for detecting muscle changes resulting from nutritional interventions (45, 46). The 12-week intervention period employed in this study also aligns with the intervention period suggested to assess functional changes (23), in line with the mean intervention period reported in the literature (42), and consistently used in real-world research (16, 47). In our study, indicators suggestive of improved function are shown. Approximately a 1.5 kg difference in handgrip strength was observed, which is comparable to the results of previous meta-analyses (1.76 kg) in (42) and (1.012 kg) in (43). These were accompanied by a significant increase (p-value>0.001) in calf circumference. This is particularly relevant since sarcopenia is common among malnourished patients and leads to substantial impairments (48). The observed increase in calf circumference exceeds the values reported in the meta-analysis evaluating ONS efficacy in community-dwelling older people (43) or in similarly aged adults receiving a powder-based ONS (34).

It is important to note that plant-based protein products may present different outcomes regarding anabolic effects compared to their animal counterparts, primarily due to differences in digestibility, amino acid composition, and the presence of limiting amino acids (49). In particular, a reduced leucine content might compromise the stimulation of muscle protein synthesis (50). Although recent evidence has shown modest increases in muscle mass with animal proteins compared to non-soy plant proteins (51), it is worth acknowledging that strategies such as plant-based protein combinations or leucine-enriched formulations may aid in achieving the nutritional quality and physiological goals (52).

### Gastrointestinal tolerance with ONS

Our study also included a notable proportion (28.7%) of patients with gastrointestinal disorders to evaluate pbONS as an alternative to the specific protein-enriched formulas traditionally recommended for these patients (53). In our study, gastrointestinal symptom scores were relatively low, indicating that both products were well tolerated, a finding consistent with previous studies on similar products (54). While there was no significant difference between the aONS and pbONS groups, pbONS patients exhibited a trend towards improved gastrointestinal symptoms compared to those receiving aONS. Although evidence on plant-based ONS remains limited, a recent narrative review suggested that vegetarian diets may provide better gastrointestinal tolerance, mainly by enhancing gut microbiota (55). Our results align with those observed by Delsoglio et al. (23), where gastrointestinal symptoms (diarrhea, constipation, vomiting, nausea, abdominal pain, and bloating) remained low after 12 weeks of pbONS intervention, and 79% of patients reported good tolerance to the supplement.

### Patients’ compliance with ONS

Compliance was high in both groups (90.9% for aONS and 82.7% for pbONS), beyond the 72.9% rate reported by Jobse et al. (47) and consistent with an earlier observational study on pbONS (94.0%) (23). High compliance is crucial for treatment effectiveness, as patients with greater adherence achieve higher body weight gain than those with poor adherence (median of 3 vs. −0.2 kg) (47). Additionally, compliance above 75% is necessary for ONS to be clinically feasible and cost-effective (56). Factors contributing to good compliance include the availability of product options to prevent flavor fatigue and meet patient preferences (20, 56). Furthermore, a literature review highlighted that ready-to-use liquid supplements in a convenient format (200 ml), such as the one employed here, are more suited and less disruptive to patients’ diets than solid supplements, resulting in higher adherence rates (56).

As mentioned above, both groups exhibited higher treatment compliance rates than previous studies. However, a statistically significant difference was observed, with patients in the pbONS group demonstrating lower compliance. We hypothesize that this finding is not related to tolerability, as gastrointestinal and patient satisfaction scores were comparable between groups. Although reduced compliance could potentially influence efficacy outcomes, the observation that patients in the pbONS group achieved similar efficacy results despite lower adherence suggests that the actual efficacy of pbONS may be equal to or greater than that observed in this study if higher compliance were attained.

### Patients’ satisfaction with ONS

Finally, this study incorporated patient opinions on ONS using *ad hoc* questionnaires. Consistent with previous findings, patients reported high overall satisfaction, particularly regarding flavor and ease of consumption, surpassing values recently reported for other products in the same range (54). Our study showed slightly higher overall and flavor satisfaction scores compared to previous pbONS evaluations (22), with mean ratings exceeding 8 out of 10. Scores above 6/10 points have been defined as indicators of good acceptability (57), which is frequently observed with plant-based products (58). This may partly be because plant-based ONS provide appetite satisfaction and satiety similar to animal-based counterparts (59). Most patients in our study reported feeling satiated after taking ONS (71.4 and 78.8%), as has been reported for similar products (54), while not significantly affecting regular diet intake. These findings support pbONS as a viable and effective alternative to animal-based ONS, further reinforced by positive patient feedback.

### Study limitations

This study has several limitations. First, while the sample is representative of real-world patients, some baseline characteristics were not fully matched between groups, potentially influencing results. Second, patient adherence to oral nutritional supplements (ONS) was self-reported, introducing potential bias. Third, variations in the timing of ONS consumption were not controlled, which may have affected outcomes. Fourth, calf circumference was used as an indicator of muscle mass because it is a simple measure widely applied in clinical practice; however, given its limited ability to fully reflect changes in body composition, adjusted calf circumference, handgrip strength, and body weight were also considered to provide a more complete assessment. Fifth, a possible limitation of this study is that a detailed assessment of dietary intake following ONS consumption was not performed. Although subjective satiety was reported by most patients, the potential impact on actual caloric and protein intake from subsequent meals was not evaluated. This impact might also be affecting the results observed in weight gain and improved status. Future studies should incorporate precise dietary intake measurements to allow a more comprehensive assessment of the nutritional impact of ONS. Additionally, the study did not assess the impact of dietary counselling or physical activity, which could influence nutritional status. Finally, as the study was conducted in Spain, findings may not be fully generalizable to other healthcare systems or populations. Moreover, this study has several strengths. It is the first randomized clinical trial comparing plant-based and animal-based oral nutritional supplements (ONS) in malnourished patients, providing valuable clinical insights. The multicenter design enhances the generalizability of the findings. Additionally, the study assessed multiple outcomes, including body weight, functional capacity, muscle mass, and gastrointestinal tolerance, offering a comprehensive evaluation. Patient adherence and satisfaction were also considered, strengthening the real-world applicability. The 12-week follow-up period allowed for meaningful assessments of nutritional improvements. Lastly, the study followed rigorous methodology, adhering to CONSORT guidelines and ethical standards, ensuring high-quality evidence.

## Conclusion

This study substantiates that plant-based oral nutritional supplements (pbONS) might be a viable alternative to traditional animal-based ONS for malnourished patients. Both formulations improved body weight and nutritional status, and also suggested possible benefits on functional status, with no significant differences between groups. Additionally, pbONS were well-tolerated, with high adherence and patient satisfaction. These findings support the use of pbONS as an effective and acceptable alternative nutritional intervention, particularly for individuals with dietary restrictions or preferences. Given the growing demand for plant-based options, this study provides valuable evidence for the possibility of integrating pbONS into routine clinical practice to optimize personalized nutrition strategies tailored to individual patient profiles. However, the results should not be generalized to the entire population and may be more applicable to specific clinical contexts or patient profiles. It is also important to consider the aforementioned limitations to fully understand the findings of the present study.

## Data Availability

The original contributions presented in the study are included in the article/Supplementary material, further inquiries can be directed to the corresponding author.

## Glossary

aONS: ONS based on ingredients from animal sources
ASPEN: American Society of Parenteral and Enteral Nutrition
BL: Baseline
BMI: Body Mass Index
COPD: Chronic obstructive pulmonary disease
CRP: C-reactive protein
DRM: Disease-Related Malnutrition
ECOG: Eastern Cooperative Oncology Group
ESMO: European Society for Medical Oncology
ESPEN: European Society for Clinical Nutrition and Metabolism
FV: Final visit
GFR: Glomerular filtration rate
GLIM: Global Leadership Initiative on Malnutrition
GSRS: Gastrointestinal Symptom Rating Scale
HbA1c: Hemoglobin A1c
HIV: Human immunodeficiency virus
MUST: Malnutrition Universal Screening Tool
ONS: Oral Nutritional Supplement
pbONS: Plant-based oral nutritional supplement
SD: Standard deviation
TSH: Thyroid-stimulating hormone
VAS: Visual analog scale
VB: Baseline visit
VF: Final visit
