# Supplementary material for: Effect of a new plant-based high-energy oral nutritional supplement in adult malnourished patients: an open-label, randomized clinical trial
Source: Front Nutr. 2025 Nov 20;12:1667954. doi: 10.3389/fnut.2025.1667954 (PMC12677066; doi:10.3389/fnut.2025.1667954)
Supplement: Supplementary file 1 [file Supplementary_file_1.docx]

**Supplementary Data**

Supplementary Figure 1 Effects of ONS on body weight changes (intention-to-treat population). (A) Body weight data by group, differences at Baseline and at Final Visit; (B) Body weight improvement; (C) Box-plot of body weight gain (%); (D) Non-inferiority in weight gain between the two study groups.

**
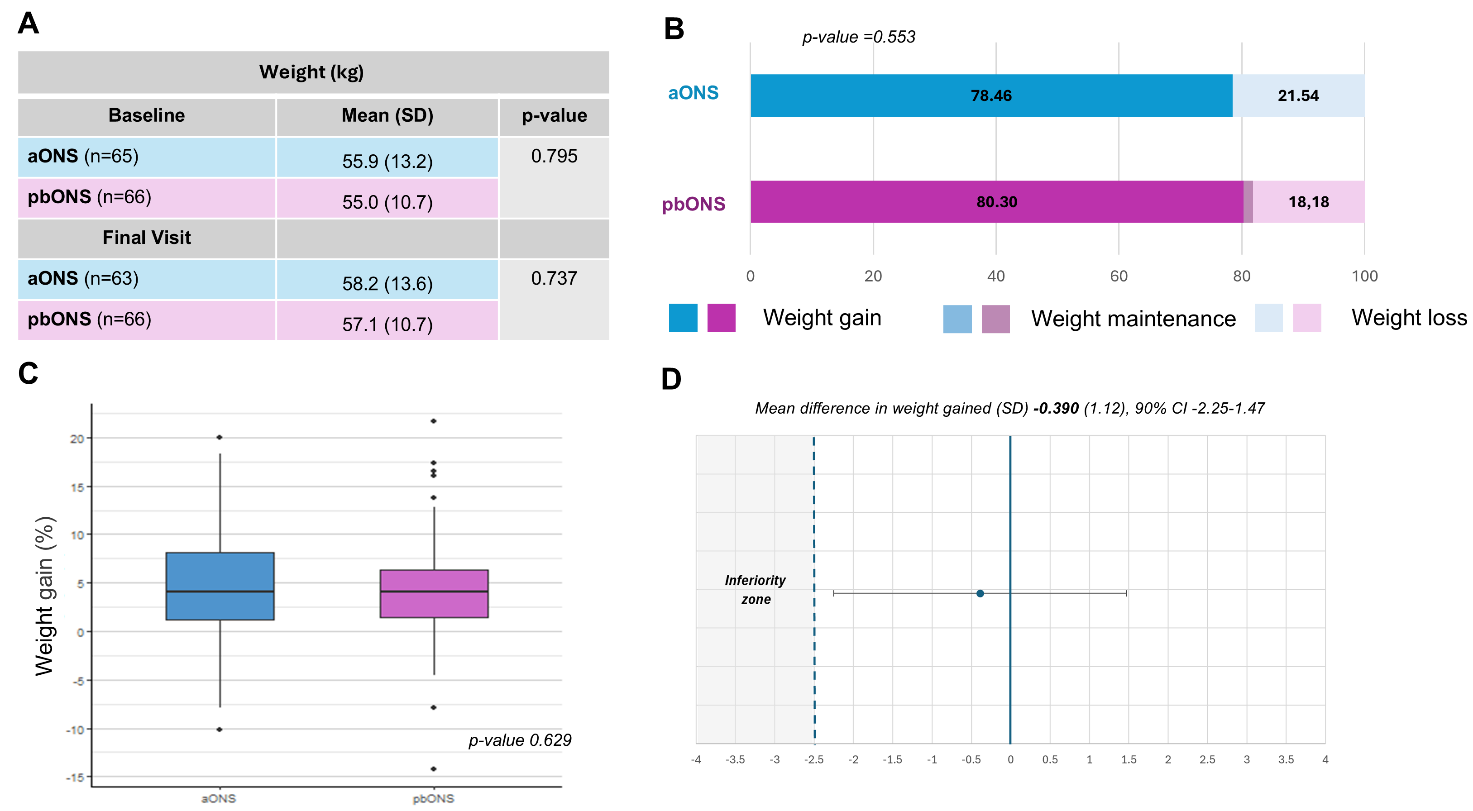
**

| **Supplementary Table 1:** Characteristics of the ONS | | |
| --- | --- | --- |
| **Variable** | **pbONS** | **aONS** |
| **Energy value (Kcal/kJ)** | 150/630 | 150/630 |
| **Fats (g)** | 5.8 | 5.8 |
| Saturated | 0.61 | 0.6 |
| **Carbohydrates (g)** | 18.6 | 18.4 |
| Sugars | 9.4 | 6.7 |
| Lactose | <0,025 | - |
| **Dietary fiber (g)** | 0.005 | 0 |
| **Proteins (g)** |  |  |
| Caseine | 6.0 | 5.59 |
| Soy and pea | 0 | 0.22 |
| **Salt (g)** | 0.25 | 0.23 |
| **Vitamins** |  |  |
| Vit. A (µg) | 93.8 | 123 |
| Vit. D (µg) | 2.69 | 1.1 |
| Vit. E (mg α-TE) | 2.45 | 1.9 |
| Vit. K (µg) | 8.75 | 8 |
| Thiamine (mg) | 0.15 | 0.23 |
| Riboflavin (mg) | 0.20 | 0.24 |
| Niacin (mg NE) | 2.13 | 2.7 |
| Pantothenic acid (mg) | 0.95 | 0.8 |
| Vit. B6 (mg) | 0.21 | 0.26 |
| Folic acid (µg) | 41.3 | 40 |
| Vit. B12 (mg) | 0.50 | 0.32 |
| Biotin (µg) | 6.40 | 6 |
| Vit. C (mg) | 13.8 | 15 |
| **Minerals and trace elements** |  |  |
| Na (mg) | 102 | 90 |
| K (mg) | 211 | 159 |
| Cl (mg) | 155 | 87 |
| Ca (mg) | 125 | 91 |
| P (mg) | 77.2 | 78 |
| Mg (mg) | 17.5 | 23 |
| Fe (mg) | 2.07 | 2.4 |
| Zn (mg) | 1.43 | 1.8 |
| Cu (mg) | 0.20 | 0.27 |
| Mn (mg) | 0.15 | 0.5 |
| F (mg) | 0.17 | 0.15 |
| Mo (µg) | 15.8 | 15 |
| Se (µg) | 8.75 | 8.6 |
| Cr (µg) | 4.38 | 10 |
| I (µg) | 18.7 | 20 |
| **Others** |  |  |
| Carotenoids (mg) | 50.0 | 0.3 |
| Choline (mg) |  | 50.0 |
| *aONS, animal-based ONS; pbONS, plant-based ONS; ONS, oral nutritional supplement* | | |

| **Supplementary Table 2:** p-values in reference between baseline and final visit | | | |
| --- | --- | --- | --- |
| **Variable** | **ONS** | **p-value** | **Statistical Test** |
| **Weight** |  |  |  |
|  | aONS | <0.001 | Wilcoxon Signed-Rank |
|  | pbONS | <0.001 |  |
| **MUST** |  |  |  |
|  | aONS | <0.001 | Wilcoxon Signed-Rank |
|  | pbONS | <0.001 |  |
| **GLIM** |  |  |  |
| Severe Malnutrition | aONS | <0.001 | Wilcoxon Signed-Rank |
|  | pbONS | <0.001 |  |
| Moderate Malnutrition | aONS | <0.001 |  |
|  | pbONS | <0.001 |  |
| **Handgrip strength, dynamometry** |  |  |  |
|  | aONS | 0.047 | Wilcoxon Signed-Rank |
|  | PbONS | 0.003 |  |
| **Calf circumference** |  |  |  |
|  | aONS | <0.001 | Wilcoxon Signed-Rank |
|  | pbONS | <0.001 |  |
| **Global risk of malnutrition** |  |  |  |
| High risk | aONS | <0.001 | McNemar |
|  | pbONS | <0.001 |  |
| Intermediate Risk | aONS | 0.414 |  |
|  | pbONS | 0.371 |  |
| Low risk | aONS | <0.001 |  |
|  | pbONS | <0.001 |  |
| **GSRS** |  |  |  |
| Reflux | aONS | 0.045 | Wilcoxon Signed-Rank |
|  | pbONS | 0.006 |  |
| Abdominal pain | aONS | 0.002 |  |
|  | pbONS | <0.001 |  |
| Indigestion | aONS | 0.042 |  |
|  | pbONS | <0.001 |  |
| Diarrhea | aONS | 0.027 |  |
|  | pbONS | 0.005 |  |
| Constipation | aONS | 0.177 |  |
|  | pbONS | 0.070 |  |
| *aONS, animal-based ONS; GLIM, global leadership initiative on malnutrition; GSRS, gastrointestinal symptom rating scale; MUST, malnutrition universal screening tool; ONS, oral nutritional supplement; pbONS, plant-based ONS* | | | |

| **Supplementary Table 3.** Laboratory parameters | | | | | | | | | | | |
| --- | --- | --- | --- | --- | --- | --- | --- | --- | --- | --- | --- |
|  |  | **Visit** | **Mean** | **SD** | **Min** | **P25** | **P50** | **P75** | **Max** | n | **Lost data** |
| **Albumin**  g/dL | aONS | BL | 4.08 | 0.54 | 2.9 | 3.7 | 4.1 | 4.6 | 5.3 | 63 | 0 |
|  |  | FV | 4.27 | 0.48 | 2.7 | 4.1 | 4.3 | 4.5 | 5.3 | 61 | 2 |
|  | pbONS | BL | 4.12 | 0.58 | 2 | 3.9 | 4.2 | 4.5 | 5 | 66 | 0 |
|  |  | FV | 4.19 | 0.44 | 3.2 | 4 | 4.3 | 4.5 | 4.9 | 65 | 1 |
| **Prealbumin**  **mg/dL** | aONS | BL | 20.27 | 6.58 | 6 | 15 | 20 | 24.5 | 40 | 63 | 0 |
|  |  | FV | 23.02 | 5.3 | 5 | 20.7 | 22.8 | 26.2 | 34.3 | 61 | 2 |
|  | pbONS | BL | 22.08 | 6.95 | 7.7 | 18 | 22 | 27.4 | 40 | 63 | 3 |
|  |  | FV | 23.07 | 7.46 | 2.4 | 19 | 22 | 28 | 40 | 62 | 4 |
| **Zinc**  **µg/ml** | aONS | BL | 5.9 | 3.45 | 0.59 | 2.15 | 7.3 | 8.55 | 11 | 36 | 27 |
|  |  | FV | 8.03 | 4.08 | 0.76 | 6.6 | 8.4 | 10.4 | 17.6 | 37 | 26 |
|  | pbONS | BL | 7.61 | 3.14 | 0.99 | 6.15 | 8.08 | 9.43 | 14.5 | 52 | 14 |
|  |  | FV | 8.75 | 3.33 | 0.01 | 7.7 | 8.81 | 10 | 19 | 54 | 12 |
| **Hemoglobin**  **g/dL** | aONS | BL | 12.96 | 1.95 | 9.7 | 11.5 | 12.8 | 14.1 | 18.4 | 63 | 0 |
|  |  | FV | 13.57 | 1.61 | 10 | 12.5 | 13.5 | 14.5 | 17.7 | 63 | 0 |
|  | pbONS | BL | 12.99 | 1.43 | 10 | 11.9 | 13 | 14.1 | 15.6 | 66 | 0 |
|  |  | FV | 13.35 | 1.57 | 10 | 12.2 | 13.4 | 14.4 | 17.3 | 65 | 1 |
| **CRP**  **mg/L** | aONS | BL | 20.03 | 34.23 | 1 | 1.6 | 4.76 | 24.1 | 190.35 | 60 | 3 |
|  |  | FV | 8.53 | 18.31 | 1 | 1 | 3.28 | 9.48 | 129 | 62 | 1 |
|  | pbONS | BL | 8.21 | 15.77 | 1 | 1 | 1.63 | 8.25 | 91.79 | 64 | 2 |
|  |  | FV | 8.14 | 16.23 | 1 | 1 | 1.56 | 6 | 73.8 | 65 | 1 |
| **Ferritin**  **ng/ml** | aONS | BL | 222.22 | 259.75 | 8.5 | 57 | 147.95 | 275 | 1,459.00 | 62 | 1 |
|  |  | FV | 210.48 | 264 | 9.8 | 52.5 | 112 | 286.1 | 1,711.40 | 60 | 3 |
|  | pbONS | BL | 186.1 | 192.29 | 8 | 36.85 | 122 | 255.5 | 801.1 | 64 | 2 |
|  |  | FV | 179.88 | 184.06 | 12 | 34 | 119.35 | 242 | 600 | 62 | 4 |
| **Transferrin**  **mg/dL** | aONS | BL | 225.33 | 46.23 | 154 | 185 | 218 | 255 | 351 | 63 | 0 |
|  |  | FV | 240.35 | 50.8 | 150 | 201 | 236 | 279.5 | 352 | 60 | 3 |
|  | pbONS | BL | 249.23 | 108.07 | 150 | 195 | 234 | 271 | 999 | 66 | 0 |
|  |  | FV | 247.06 | 54.37 | 150 | 215 | 236 | 274 | 376 | 65 | 1 |
| **HbA1c***  **%** | aONS | BL | 5.49 | 0.5 | 3.8 | 5.2 | 5.5 | 5.8 | 6.7 | 63 | 0 |
|  |  | FV | 5.55 | 0.49 | 4.4 | 5.2 | 5.6 | 5.9 | 6.5 | 62 | 1 |
|  | pbONS | BL | 5.53 | 0.66 | 4 | 5.2 | 5.5 | 5.8 | 7.9 | 66 | 0 |
|  |  | FV | 5.7 | 0.77 | 4 | 5.3 | 5.6 | 6.05 | 8 | 64 | 2 |
| **Cholesterol**  **mg/dL** | aONS | BL | 183.44 | 48.97 | 93 | 152 | 176.5 | 213 | 303 | 62 | 1 |
|  |  | FV | 184.15 | 40.67 | 68 | 160 | 180 | 219 | 266 | 61 | 2 |
|  | pbONS | BL | 171.45 | 46.21 | 68 | 140 | 170 | 201 | 299 | 66 | 0 |
|  |  | FV | 175.67 | 46.47 | 97 | 146 | 170 | 196 | 393 | 64 | 2 |
| **creatinine****  **mg/dL** | aONS | BL | 0.75 | 0.21 | 0.3 | 0.62 | 0.72 | 0.83 | 1.5 | 63 | 0 |
|  |  | FV | 0.82 | 0.33 | 0.44 | 0.65 | 0.71 | 0.84 | 2.52 | 62 | 1 |
|  | pbONS | BL | 0.78 | 0.23 | 0.4 | 0.6 | 0.75 | 0.9 | 1.41 | 66 | 0 |
|  |  | FV | 0.82 | 0.26 | 0.42 | 0.64 | 0.75 | 0.93 | 1.6 | 65 | 1 |
| **TSH*****  **mUI/L** | aONS | BL | 1.8 | 1.06 | 0.09 | 1.03 | 1.74 | 2.36 | 4.7 | 62 | 1 |
|  |  | FV | 2.11 | 1.42 | 0.38 | 1.15 | 1.73 | 2.6 | 7.05 | 58 | 5 |
|  | pbONS | BL | 2.21 | 1.59 | 0.37 | 1.36 | 1.79 | 2.57 | 9.49 | 65 | 1 |
|  |  | FV | 2.35 | 1.74 | 0.37 | 1.26 | 1.81 | 2.98 | 9.99 | 65 | 1 |
| **GFR**  **ml/min/1,73 m2** | aONS | BL | 88.25 | 13.66 | 43 | 84 | 90 | 93 | 120 | 63 | 0 |
|  |  | FV | 85.88 | 17.23 | 33 | 79 | 90 | 94.84 | 120 | 61 | 2 |
|  | pbONS | BL | 84.33 | 16.32 | 35 | 83 | 90 | 90 | 120 | 66 | 0 |
|  |  | FV | 82.62 | 16.84 | 33 | 76.5 | 90 | 90 | 120 | 64 | 2 |
| **Lymph**  **lymph/mm³** | aONS | BL | 2,029.84 | 1,131.29 | 550 | 1,250.00 | 1,770.00 | 2,300.00 | 7,000.00 | 63 | 0 |
|  |  | FV | 2,140.16 | 1,098.42 | 530 | 1,300.00 | 1,800.00 | 2,820.00 | 6,000.00 | 63 | 0 |
|  | pbONS | BL | 2,216.23 | 1,772.69 | 500 | 1,150.00 | 1,745.00 | 2,370.00 | 9,000.00 | 66 | 0 |
|  |  | FV | 2,092.89 | 993.54 | 640 | 1,390.00 | 1,910.00 | 2,660.00 | 4,460.00 | 65 | 1 |
| *aONS, animal-based ONS; BL, baseline; CRP, c-reactive protein; FV, final visit; GFR, glomerular filtration rate; HbA1c, glycosylated hemoglobin; Lymph, lymphocytes; ONS, oral nutritional supplement; pbONS, plant-based ONS; SD, standard deviation; TSH, thyroid-stimulating hormone* | | | | | | | | | | | |
